# The transcriptional repressor Blimp1 is expressed in rare luminal progenitors and is essential for mammary gland development

**DOI:** 10.1242/dev.136358

**Published:** 2016-05-15

**Authors:** Mohammed I. Ahmed, Salah Elias, Arne W. Mould, Elizabeth K. Bikoff, Elizabeth J. Robertson

**Affiliations:** Sir William Dunn School of Pathology, University of Oxford, Oxford OX1 3RE, UK

**Keywords:** Blimp1, Prdm1, Transcriptional repressor, Mammary gland morphogenesis, Luminal progenitors, Polarity, Lumen formation, 3D culture, Mouse

## Abstract

Mammary gland morphogenesis depends on a tight balance between cell proliferation, differentiation and apoptosis, to create a defined functional hierarchy within the epithelia. The limited availability of stem cell/progenitor markers has made it challenging to decipher lineage relationships. Here, we identify a rare subset of luminal progenitors that express the zinc finger transcriptional repressor Blimp1, and demonstrate that this subset of highly clonogenic luminal progenitors is required for mammary gland development. Conditional inactivation experiments using K14-Cre and WAPi-Cre deleter strains revealed essential functions at multiple developmental stages. Thus, Blimp1 regulates proliferation, apoptosis and alveolar cell maturation during puberty and pregnancy. Loss of Blimp1 disrupts epithelial architecture and lumen formation both *in vivo* and in three-dimensional (3D) primary cell cultures. Collectively, these results demonstrate that Blimp1 is required to maintain a highly proliferative luminal subset necessary for mammary gland development and homeostasis.

## Abstract (precis)

**Highlighted article:** In the mouse mammary gland, Blimp1 marks a rare progenitor population, and is required for cell proliferation and polarity as well as efficient milk production.

## INTRODUCTION

The mammary gland, a defining feature of mammals, is a highly specialized organ that produces and delivers milk from the mother to the newborn. The resulting nutritional benefits and postnatal immune protection are credited with the evolutionary success of mammals. Its orchestrated morphogenesis during puberty, pregnancy, lactation and involution make the mammary gland a powerful model system for studying the genetic circuits controlling epithelial growth and development. Mammary glands initially become visible at around embryonic day (E) 11.5 as placode-like structures that progressively invade the underlying mesenchyme, the so-called mammary fat pad, to form an epithelial tree (Hennighausen and Robinson, 2005). The epithelium is composed of two distinct populations: the outer myoepithelial/basal cells and the inner luminal cells. Extensive elongation and branching of the ducts via the terminal end buds (TEBs) during puberty generates the mature ductal network. Subsequent expansion during pregnancy in response to steroid hormones gives rise to highly specialized milk-producing alveoli. During the suckling-to-weaning transition, the alveoli undergo a process of involution leading to regression of the gland (Visvader and Stingl, 2014).

Mammary stem cells (MaSCs) within the epithelium are responsible for its striking regenerative capacity upon successive rounds of pregnancy (Hoshino and Gardner, 1967; Smith and Medina, 1988). MaSCs, sharing characteristics of basal cells, lack steroid hormone receptors (Asselin-Labat et al., 2010; Shackleton et al., 2006; Stingl et al., 2006). The striking morphological changes during pregnancy are triggered in response to RANKL (also known as Tnfsf11) paracrine signalling activated by adjacent oestrogen and progesterone-responsive (ERα^+^PR^+^; also known as Esr1 and Pgr, respectively) luminal cells (Asselin-Labat et al., 2010; Joshi et al., 2010). Recent studies have identified several distinct stem and progenitor cell subpopulations (Shackleton et al., 2006; Shehata et al., 2012; Stingl et al., 2006; Villadsen et al., 2007). The cell fate choices made during mammary gland development have been extensively studied, but the precise details of lineage commitment remain controversial. Both unipotent and bipotent progenitors giving rise to luminal and/or myoepithelial basal subpopulations have been identified in lineage-tracing experiments (Van Keymeulen et al., 2011; van Amerongen, et al., 2012; Prater et al., 2014; Rios et al., 2014; Wang et al., 2015). The heterogeneous luminal compartment is known to contain several distinct cell subpopulations that display diverse differentiation states and proliferative capacities (Lafkas et al., 2013; Rodilla et al., 2015; Sale et al., 2013; Shehata et al., 2012; Sleeman et al., 2007). A subset of ERα^+^ luminal cells considered to be functionally mature rarely proliferate in adult mammary tissue (Russo et al., 1999; Shehata et al., 2012). By contrast, ERα^−^ luminal cells that robustly express the Ets transcription factor Elf5 function as highly proliferative alveolar progenitors (Oakes et al., 2008; Rios et al., 2014; Rodilla et al., 2015; Sleeman et al., 2007). This high degree of cellular heterogeneity, in combination with the limited availability of stem/progenitor markers, has made it challenging to define lineage hierarchies.

The zinc finger transcriptional repressor B-lymphocyte-induced maturation protein 1 (Blimp1; also known as Prdm1), originally cloned as negative regulator of β-interferon gene expression (Keller and Maniatis, 1991) and subsequently identified as a master regulator of plasma cell terminal differentiation (Martins and Calame, 2008; Shapiro-Shelef et al., 2003), governs cell fate decisions in the developing embryo and adult tissues (Bikoff et al., 2009). In B cells and macrophages, Blimp1 represses *Myc* expression to arrest cellular proliferation (Martins and Calame, 2008), whereas in activated T lymphocytes, Blimp1 targets the cytokine IL-2 to block cell proliferation and promote effector T-cell maturation (Martins et al., 2008). In the early embryo, Blimp1 is required to specify the primordial germ-cell lineage (Ohinata et al., 2005; Vincent et al., 2005), and at later developmental stages Blimp1 activities are essential for morphogenesis of the pharynx, forelimbs and placenta (Mould et al., 2012; Robertson et al., 2007). In the skin, Blimp1 maintains tissue homeostasis and epithelial barrier function (Kretzschmar et al., 2014; Magnusdottir et al., 2007). Blimp1 regulates the developmental switch responsible for postnatal reprogramming of the intestinal epithelium (Harper et al., 2011; Muncan et al., 2011). Recent studies demonstrate that Blimp1 functions as a gatekeeper in opposition to Irf1 to prevent premature activation of the MHC class I pathway in the fetal enterocytes and to maintain tolerance in the neonatal intestine in the first few weeks after birth, during colonization of the intestinal tract by commensal microorganisms (Mould et al., 2015). In human breast cancer cell lines, Blimp1 functions downstream of TGFβ1, RelB and Ras signalling to induce epithelial-mesenchymal transition (EMT) (Romagnoli et al., 2012; Wang et al., 2009). Blimp1 contributions during normal mammary gland development and tissue homeostasis have yet to be investigated.

Here, we demonstrate that Blimp1 expression marks a subset of Elf5^+^ERα^−^PR^−^ luminal-alveolar progenitors, primed in response to pregnancy hormones. Blimp1 function is essential for ductal morphogenesis during puberty and lobuloalveolar maturation during late pregnancy and lactation. Conditional inactivation disrupts the ability of luminal cells to polarize properly, leading to defective milk secretion. Collectively, these findings demonstrate for the first time that Blimp1 plays an essential role in controlling mammary gland development.

## RESULTS

### Developmentally regulated Blimp1 expression is restricted to the luminal compartment

**Fig. 1.**
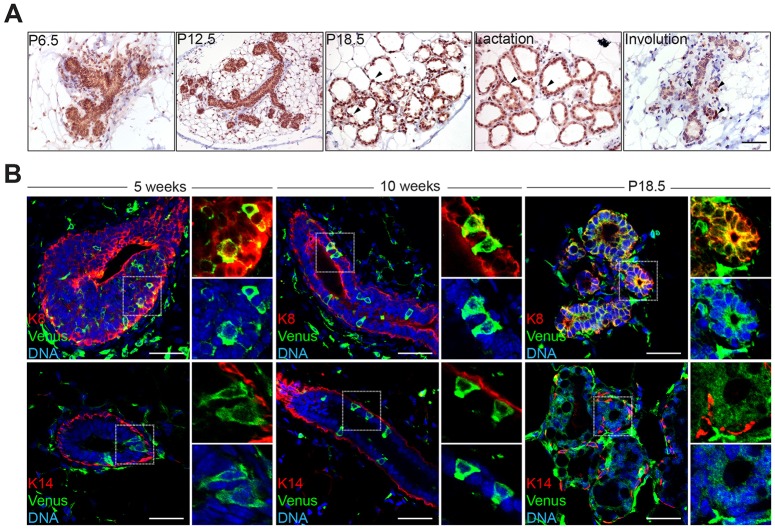
**Blimp1 expression is restricted to the luminal cell compartment of the developing mammary gland.** (A) Blimp1 immunohistochemical staining of mammary gland tissue during pregnancy (P6.5, P12.5 and P18.5) and at day 1 of lactation and day 5 of involution. During pregnancy, lactation and involution, Blimp1^+^ cells are confined to the luminal cell compartment of alveolar structures (arrowheads). (B) Cryosections from 5- and 10-week-old virgins and P18.5 Blimp1-Venus (BV) mammary glands stained for GFP (green) and Krt8 (K8; red) or Krt14 (K14; red). Blimp1-expressing cells (green) are Krt8^+^ and Krt14^−^. High-magnification images of the boxed areas are shown to the right of each image. Scale bars: 50 μm.

Western blot experiments have demonstrated that Blimp1 expression in mammary gland tissue is robustly activated during pregnancy (Romagnoli et al., 2012). To characterize Blimp1^+^ cell populations, we performed immunostaining experiments. At day 6 of pregnancy (P6.5) we observed Blimp1^+^ cells localized within the luminal compartment of epithelial structures (Fig. 1A). The highest numbers of Blimp1^+^ cells were present in the alveolar structures during late pregnancy and lactation. During involution, Blimp1 expression is confined to a small number of luminal cells within the regressing epithelium (Fig. 1A). Scattered Blimp1^+^ cells are also detectable within the stromal population (Fig. 1A). qRT-PCR analysis of basal (Lin^−^CD24^low^CD49F^high^*Krt14^+^*) and luminal (Lin^−^CD24^high^CD49F^low^*Krt8^+^*) (CD49F is also known as Itga6) subpopulations isolated from 12-week-old virgin and P18.5 mammary tissue using flow cytometry (Fig. S1A,B) confirmed that Blimp1 expression is restricted to the luminal compartment (Fig. S1C). Next, we exploited a BAC transgenic reporter strain expressing membrane Venus (mVenus) under control of the Blimp1 regulatory elements (Ohinata et al., 2008). In pubertal (5 weeks of age), and mature (10 weeks of age) virgins, a small subset of Blimp1-Venus^+^ (BV^+^) cells were visible in the TEBs, with fewer BV^+^ cells detected in the mature virgin mammary epithelium (Fig. 1B). Immunofluorescence experiments demonstrate that BV^+^ cells selectively co-expressed the luminal marker Krt8 and not the basal marker Krt14. Increased numbers of BV^+^ cells were present in alveolar structures during pregnancy (Fig. 1B).

### Blimp1 expression marks a rare subset of luminal progenitors

**Fig. 2.**
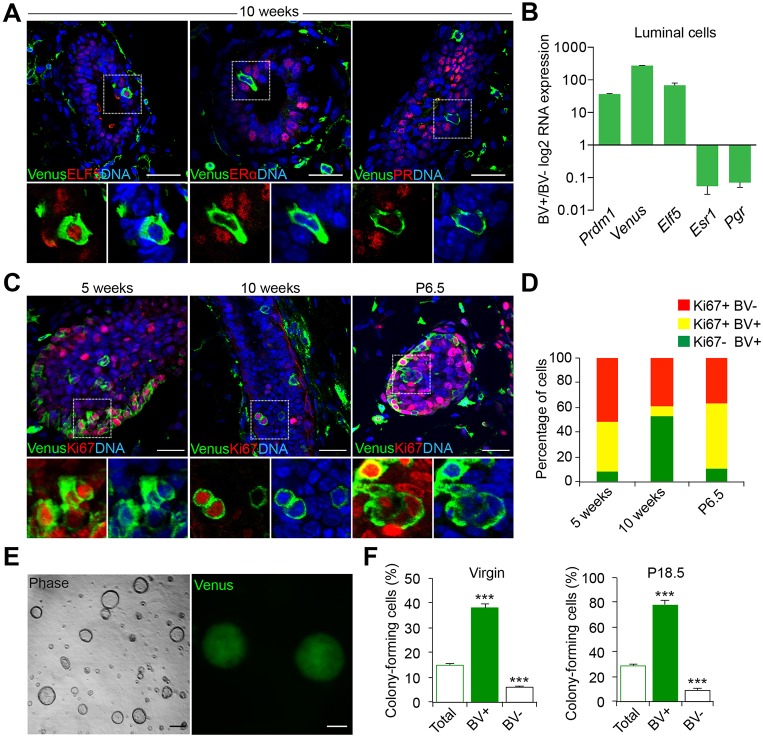
**Blimp1 expression marks highly clonogenic luminal progenitors.** (A) Cryosections from 10-week-old virgin BV mammary glands stained for GFP and Elf5, ERα or PR. Blimp1-expressing cells (green) are Elf5^+^ and ERα^−^/PR^−^. (B) FACS-sorted BV^+^ luminal cells isolated from 12-week-old virgin BV mice were analysed by qRT-PCR for *Elf5*, *Esr1* and *Pgr* expression. The values were normalized to *Hprt* expression. Data are presented as mean±s.e.m. from *n*=3 independent experiments. (C) Cryosections from 5- and 10-week-old virgin (TEBs and ductal structures) and P6.5 (alveoli) BV mice stained for GFP (green) and Ki67 (red). High-magnification images of the boxed areas in A,C are shown below each image. (D) Histogram showing the percentages of Ki67^+^BV^−^ (red), Ki67^+^BV^+^ (yellow) and Ki67^−^BV^+^ (green) at 5 and 10 weeks of age and at P6.5. BV^+^ cells are highly proliferative in 5-week TEBs and P6.5 alveoli, but display reduced proliferation in 10-week ducts. Data are presented as mean±s.e.m. from *n*=3 independent experiments. (E) Representative images of colonies formed by BV^+^ luminal cells isolated from 12-week-old virgin mice (*n*=3 independent experiments). (F) Colony-forming efficiency of BV^+^, BV^−^ and unsorted luminal cells (total) from 12-week-old virgin and P18.5 mice. BV^+^ luminal cells form significantly higher numbers of colonies compared with BV^−^ and unsorted luminal cells. Data are presented as mean±s.e.m. from *n*=3 independent experiments. ***P<0.001. Scale bars: 50 μm.

By flow cytometry we identified two distinct BV^+^ cell subpopulations (Fig. S1). As expected (Arulanandam et al., 2015; Vincent et al., 2005), Blimp1 is robustly expressed in CD31^+^ (also known as Pecam1) endothelial cells. BV^+^Lin^+^CD24^−^CD49F^−^ endothelial cells represent 2.8% and 2.4% of total dissociated cells in 12-week-old virgin and P18.5 pregnant mammary glands, respectively (Fig. S1E). Additionally, we observe a rare population of BV^+^Lin^−^CD24^high^CD49F^low^ luminal cells comprising 0.44% and 0.48% of total dissociated cells, respectively (Fig. S1D,E). *In situ* experiments demonstrate that these BV^+^ luminal cells express Elf5 but lack ERα and PR (Fig. 2A,B), suggesting that they correspond to a previously described subset of luminal progenitors (Shehata et al., 2012). To examine the proliferative status of these BV^+^ luminal cells, we assessed Ki67 (also known as Mki67) expression. In mature virgin epithelium, the majority of BV^+^ cells are quiescent (Fig. 2C). However, during puberty and at P6.5, representation of double-positive BV^+^ Ki67^+^ cells is dramatically increased (Fig. 2C,D).

To investigate directly their proliferative capabilities, sorted BV^+^ luminal cells recovered from 12-week-old virgin and P18.5 pregnant females were tested in three-dimensional (3D) Matrigel cultures (Fig. 2E,F). The colony-forming efficiencies (2.6- and 3.2-fold, respectively) of the BV^+^ cell fraction were markedly enriched compared with the total starting luminal cell population (Fig. 2F). Thus, ∼40% of plated BV^+^ luminal cells from 12-week-old virgins formed colonies compared with 14% of the starting luminal cell population (Fig. 2F). Moreover ∼80% of the BV^+^ luminal cells from P18.5 mammary glands formed colonies compared with ∼25% of the total cell population. By contrast, however, only ∼6% and ∼9% of BV^−^ luminal cells isolated at P18.5 and 12 weeks of age, respectively, formed colonies, indicating that this cell population has much-reduced clonogenic potential (Fig. 2F). Thus, BV^+^ luminal cells potentially represent a highly proliferative progenitor cell population that markedly expands during pregnancy.

### Blimp1 expression becomes dramatically upregulated in response to steroid hormones

**Fig. 3.**
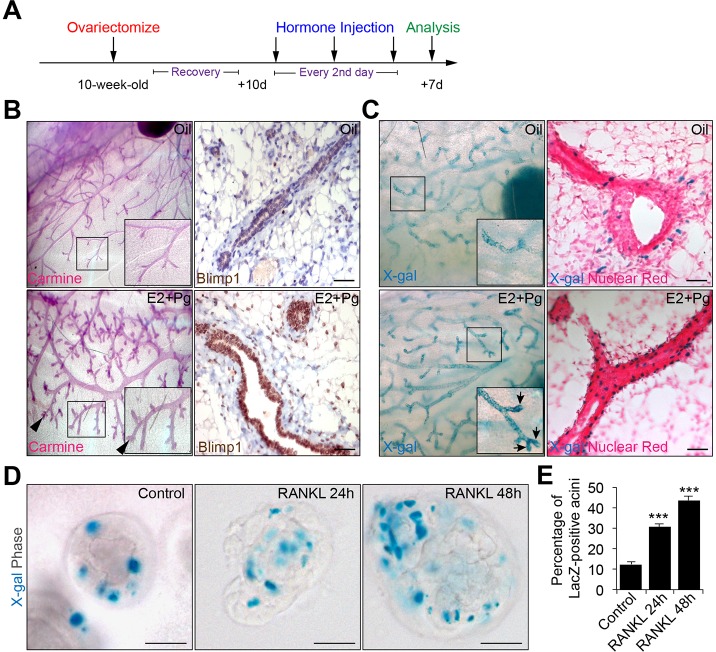
**Expansion of Blimp1^+^ luminal cells in response to hormone treatment.** (A) Wild-type and *Prdm1^Cre.IRES.nLacZ^* 10-week-old virgin mice were ovariectomized, allowed to recover for 10 days (d) and injected over 6 days with a cocktail of 17β-oestradiol and progesterone (E2+Pg) or mineral oil as a control. Mammary glands were recovered for analysis 7 days later. (B) Whole-mount Carmine staining and Blimp1 immunostaining reveal mammary gland expansion and enhanced Blimp1 expression in response to E2+Pg treatment. Arrowheads indicate the formation of alveolar buds (images representative from two independent experiments; *n*=5 mice per treatment). (C) X-gal-stained whole-mount and tissue sections of control (oil) or E2+Pg-treated *Prdm1^Cre.IRES.nLacZ^* mammary glands. Sections were counterstained with Nuclear Fast Red. Arrows indicate increased numbers of Blimp1-*lacZ*^+^ cells in the alveolar buds (images representative from two independent experiments; *n*=5 mice per treatment). Insets in B,C show high-magnification images of the boxed areas. (D) RANKL treatment results in significantly increased numbers of Blimp1-*lacZ*^+^ cells. Day 6 3D MECs from *Prdm1^Cre.IRES.nLacZ^* P15.5-P16.5 females were treated with RANKL (24 h or 48 h) prior to staining. (E) Percentage of Blimp1-*lacZ*^+^ acini in control versus RANKL-treated cultures (control: *n*=30 acini; RANKL 24 h: *n*=30 acini; RANKL 48 h: *n*=30). Error bars represent s.e.m. ***P<0.001. Scale bars: 50 μm.

Next, we treated ovariectomized wild-type females or females carrying the *Prdm1^Cre.IRES.nLacZ^* reporter allele (Mould et al., 2012) with a cocktail of 17β-oestradiol (E2) and progesterone (Pg). As expected, E2+Pg hormone injections resulted in mammary gland expansion and the formation of lobuloalveolar structures (Fig. 3B). X-gal staining and immunostaining reveal markedly increased Blimp1 expression in the ducts and nascent alveolar buds (Fig. 3B,C). Similarly, RANKL treatment in 3D cultures of primary mammary epithelial cells (MECs) from pregnant *Prdm1^Cre.IRES.nLacZ^* reporter mice results in increased numbers of Blimp1-*lacZ*^+^ cells (Fig. 3D,E). Thus, Blimp1 expression marks progenitors that proliferate in response to steroid hormones to generate the alveolar structures.

### Blimp1 functional requirements during ductal morphogenesis, lumen formation and alveologenesis

**Fig. 4.**
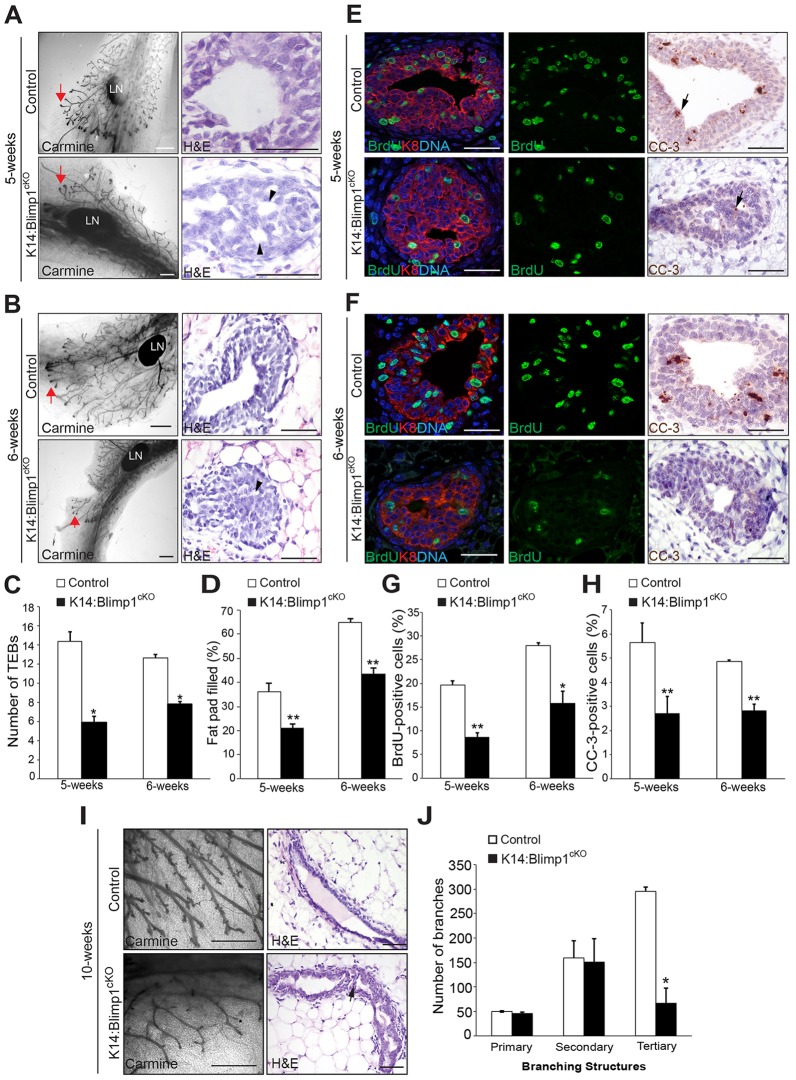
**Blimp1 conditional inactivation results in defective mammary gland morphogenesis.** (A,B) Whole-mount Carmine-stained and histological sections of mammary glands from 5- and 6-week-old virgin K14:Blimp1^cKO^ or littermate controls (representative images from *n*=3 mice of each genotype). Red arrows indicate TEBs. Black arrowheads indicate defective lumen formation within the TEBs. (C,D) Histograms showing the number of TEBs (C), and the percentage of the fat pad filled by the invading mammary epithelial front (D) past the mid-point of the lymph node, assessed by whole-mount staining. Histograms are presented as mean±s.e.m. from *n*=3 mice of each genotype. *P<0.05; **P<0.01. (E,F) Decreased proliferation and apoptosis. TEBs from 5- and 6-week-old Blimp1-deficient virgins stained for BrdU (green) and Krt8 (K8; red), or cleaved caspase-3 (CC-3) show markedly reduced numbers of BrdU^+^ and CC-3^+^ cells (arrows) (representative images from *n*=3 mice of each genotype). (G,H) Significantly reduced percentages of BrdU^+^ (G) and CC-3^+^ (H) cells within TEBs from 5- and 6-week-old virgin *Blimp1* mutants. Histograms are presented as mean±s.e.m. from *n*=3 mice of each genotype. (I) Whole-mount Carmine-stained and histological sections of mammary glands from 10-week-old virgin K14:Blimp1^cKO^ or littermate controls (representative images from *n*=3 mice of each genotype). Arrow indicates defective lumen formation within ducts. (J) Histograms showing the number of primary, secondary and tertiary branches within 10-week-old mammary glands from K14:Blimp1^cKO^ and control littermates. *P<0.05; **P<0.01. LN, lymph node. Scale bars: 1 mm (whole mount); 50 μm (sections).

To investigate Blimp1 functional requirements during ductal morphogenesis, we used a *Blimp1* conditional allele (Shapiro-Shelef et al., 2003), in combination with the K14-Cre deleter strain, termed here K14:Blimp1^cKO^. As expected, this strategy eliminates Blimp1 expression in the skin and the entire mammary gland epithelium (Fig. S2A). As judged by reduced numbers of TEBs and ductal branches (Fig. 4A-C), *Blimp1* mutant females display defective ductal morphogenesis during puberty (5 and 6 weeks of age). Mature virgin (10 weeks of age) mutant glands have significantly fewer tertiary branches (Fig. 4I,J). We observed impaired lumen formation (Fig. 4A-D,I), together with significantly reduced numbers of bromodeoxyuridine (BrdU) and cleaved caspase-3 positive (CC-3^+^) cells in the TEBs (Fig. 4E-H). Thus, loss of Blimp1 disrupts proliferation of the TEB luminal cell compartment and delays apoptosis-mediated clearing of intraluminal cells.

**Fig. 5.**
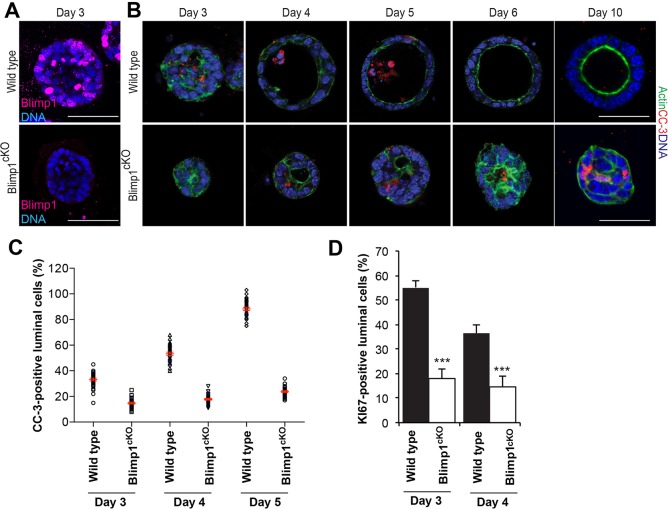
**Loss of Blimp1 delays lumen formation in MEC 3D cultures.** (A) Immunostaining of day 3 wild-type and *Prdm1^BEH/CA^;ROSA26:Cre^ERT2^* (Blimp1^cKO^) MEC 3D acini for Blimp1. Treatment with 100 nM 4OHT (to induce Cre) results in elimination of Blimp1 in Blimp1^cKO^ acini (representative images from *n*=3 independent experiments). (B) Immunostaining at day 3, 4, 5, 6 and 10 of wild-type and Blimp1^cKO^ MEC 3D acini for CC-3^+^ (red) and actin (green) (representative images from three independent experiments). Loss of Blimp1 results in altered apoptosis-mediated clearing of intraluminal cells. (C) Percentage of CC-3^+^ luminal cells. Data are presented as mean±s.e.m. values from day 3 (wild type: *n*=30 acini; Blimp1^cKO^: *n*=31 acini); day 4 (wild type: *n*=33 acini; Blimp1^cKO^: *n*=30 acini); and day 5 (wild type: *n*=32 acini; Blimp1^cKO^: *n*=32 acini) 3D cultures. (D) Percentage of Ki67^+^ luminal cells was significantly reduced in Blimp1^cKO^ MECs at day 3 and 4 compared with wild type. Data are presented as mean±s.e.m. values from day 3 (wild type: *n*=30 acini; Blimp1^cKO^: *n*=30 acini) and day 4 (wild type: *n*=30 acini; Blimp1^cKO^: *n*=30 acini) 3D culture. Error bars represent s.e.m. ***P<0.001. Scale bars: 50 μm.

Next, we tested the ability of primary MECs isolated from P15.5-P16.5 mammary glands to form acini in 3D cultures (Akhtar and Streuli, 2013). Blimp1^cKO^ MECs were generated from *Prdm1^CA/CA^* females harbouring the *ROSA26:CreERT2* allele (Vooijs et al., 2001) and treated *in vitro* with 4-hydroxy-tamoxifen (4OHT) to induce Cre activity and eliminate Blimp1 expression (Fig. 5A). CC-3^+^ intraluminal cells were initially visible in wild-type cultures at day 3 (Fig. 5B), and the proportion of CC-3^+^ cells increased over time. By day 6, complete clearing of the central lumen resulted in the formation of cellular spheres that display normal apicobasal polarity (Fig. 5B,C). By contrast, highly disorganized Blimp1-depleted acini displayed reduced numbers of Ki67^+^ cells and intraluminal CC-3^+^ cells, and failed to acquire correct apicobasal polarity (Fig. 5). At day 10, mutant acini contain a high proportion of CC-3^+^ cells, but still lack a distinct lumen (Fig. 5B). These *in vitro* results recapitulate the reduced proliferative potential and apoptosis-mediated luminal clearing defects observed *in vivo* (Fig. 4; Fig. S3A,B).

To examine Blimp1 functional requirements at later stages during pregnancy and lactation, we crossed the conditional allele with the WAPi-Cre deleter strain (Fig. S4). The WAPi-Cre transgene, initially expressed during pregnancy, is exclusively localized to the luminal compartment (Wintermantel et al., 2002). At P12.5, as for the K14:Blimp1^cKO^, in WAP:Blimp1^cKO^ glands, alveologenesis was severely compromised and numerous aberrant TEB-like structures reminiscent of collapsed alveoli were detectable (Fig. S4A,B). ErbB2 expression, required for normal mammary development and lactation through activation of Nrg1 (Jones and Stern, 1999), was markedly reduced (Fig. S4C,D). Additionally, the percentage of ERα^+^ cells was markedly elevated (Fig. S4E,F). At late pregnancy, mutant glands display reduced branching and small, sparsely distributed alveoli that were highly vacuolated (Fig. S4G,H). Thus, we conclude that Blimp1 function is required to promote lumen formation and lobuloalveolar expansion.

### Blimp1 inactivation causes epithelial cell polarity defects during puberty, pregnancy and lactation

**Fig. 6.**
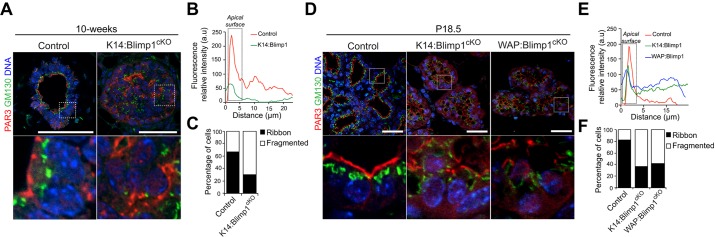
**Blimp1 inactivation causes epithelial cell polarity defects during puberty, pregnancy and lactation.** (A) Representative images of 10-week-old mammary glands from K14:Blimp1^cKO^ and control littermates stained for GM130 (green) and Par3 (red) (*n*=3 mice of each genotype). (B) Representative line-scan analysis (relative fluorescence intensity; minimum of 20 cells were analysed per genotype), reveals a failure of Par3 apical accumulation in Blimp1-deficient ductal cells. (C) Percentages of ductal cells showing ribbon-like and fragmented localization of GM130 Golgi in control and *Blimp1*-mutant mammary glands. (D) Representative images of mammary glands (P18.5) from K14:Blimp1^cKO^, WAP:Blimp1^cKO^ and control littermates stained for GM130 (green) and Par3 (red) (*n*=3 mice of each genotype). High-magnification images of the boxed areas in A,D are shown below. (E) Representative line-scan analysis (relative fluorescence intensity; minimum of 20 cells were analysed per genotype), reveals lack of Par3 apical accumulation in Blimp1-deficient alveolar cells. (F) Percentages of alveolar cells showing ribbon-like and fragmented localization of GM130 Golgi in control and *Blimp1*-mutant mammary glands. Scale bars: 50 μm.

To investigate epithelial cell polarity defects during mammary gland development, we examined localization of Par3 and the *cis*-Golgi matrix protein 130 (GM130; also known as Golga2) (Fig. 6). As expected, in control glands, Par3 localizes at tight junctions and the apical surface of luminal cells. By contrast, in both mutant virgin duct epithelia (Fig. 6A-C) and P18.5 alveoli (Fig. 6D-F), Par3 staining is more diffuse, and accumulates in the cytoplasm. Similarly, in control epithelia, GM130 was predominantly localized in an apical position facing the lumen. However, in striking contrast, in Blimp1-deficient luminal cells GM130 was dispersed within the cytoplasm and lacked its characteristic polarized distribution.

**Fig. 7.**
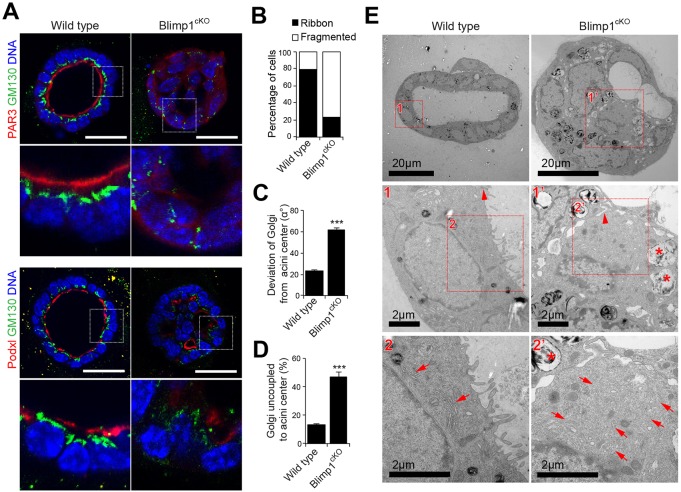
**Loss of Blimp1 alters lumen formation and maturation in MEC 3D cultures.** (A) Wild-type and *Prdm1^CA/CA^:ROSA26:CreERT2* (Blimp1^cKO^) day 6 4OHT-treated MEC 3D acini stained for GM130 (green) and Par3 (red; top panel) or podocalyxin (Podxl, red; bottom panel). MECs were treated with 4OHT for 24 h following plating. Scale bars: 50 μm. (B) Percentage of acini with ribbon-like and fragmented localization of GM130 Golgi in wild-type and Blimp1^cKO^ day 6 4OHT-treated MEC 3D acini (wild type: *n*=30 acini; Blimp1^cKO^: *n*=30 acini). (C) Deviation of the Golgi from acini centre (α°) (wild type: *n*=30 acini; Blimp1^cKO^: *n*=30 acini). (D) Percentage of acini with Golgi uncoupled to centre (wild type: *n*=30 acini; Blimp1^cKO^: *n*=30 acini). Data are presented as mean±s.e.m. ***P<0.001. (E) Representative electron micrographs of wild-type and Blimp1^cKO^ day 6 4OHT-treated MEC 3D acini revealing alterations in the establishment of an apical-basal polarity in mutant acini compared with wild type, accompanied by dismantled tight junctions (1′ vs 1; arrowheads), fragmentation of the Golgi apparatus (2′ vs 2; arrows) and loss of apical villi.

Blimp1 inactivation in 3D MEC cultures via 4OHT treatment similarly resulted in loss of luminal cell polarity. Thus, Par3 and podocalyxin (Podx1) failed to accumulate at the apical surface (Fig. 7A-D). Next, we used transmission electron microscopy (TEM) to characterize the ultrastructural defects in Blimp1^cKO^ day 6 3D acini (Fig. 7E). As expected, wild-type MECs display an apical localization of the Golgi apparatus, and differentiated tight junctions and microvilli, consistent with their mature apical surface. In striking contrast, Blimp1^cKO^ cells failed to develop tight junctions or microvilli, and instead contained high numbers of large endosome-, lysosome- and autophagosome-like structures. Moreover, the Golgi apparatus was fragmented throughout the cytosol of Blimp1^cKO^ MECs. Thus, Blimp1 is essential to maintain the alveolar secretory machinery.

**Fig. 8.**
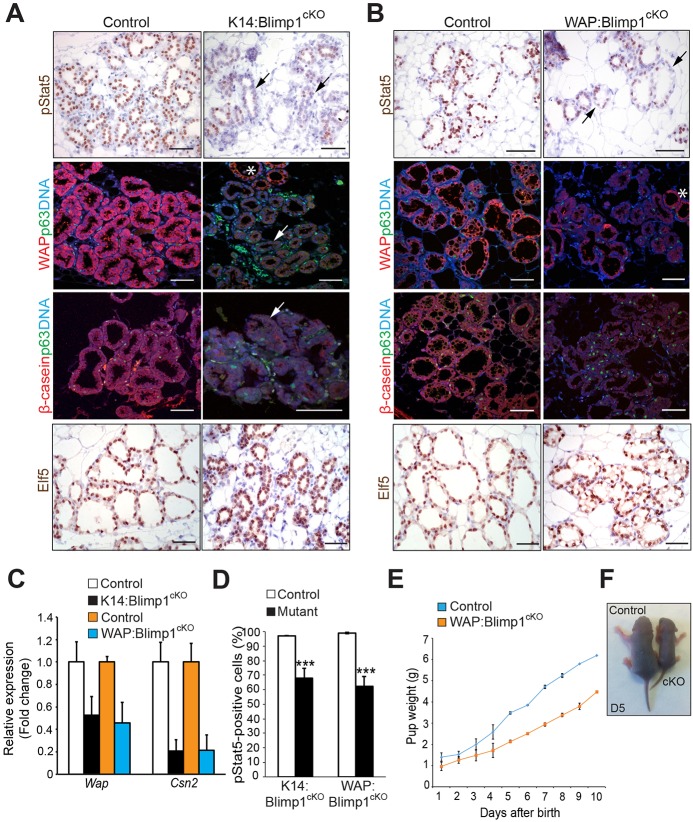
**Blimp1 function is essential for alveolar maturation during pregnancy and lactation.** (A,B) Representative images of mammary glands (P18.5) stained for pStat5, p63 (green), Wap (red) or β-casein (red), and Elf5 (representative images from *n*=3 mice of each genotype); black arrows indicate a dramatic reduction of pStat5^+^ cells and white arrows indicate markedly reduced expression of milk proteins Wap and β-casein in mutant alveoli. Asterisks indicate areas showing normal expression levels of Wap. The numbers of p63^+^ basal cells are similar in control and mutant tissues. (C) qRT-PCR analysis demonstrates markedly reduced expression of *Csn2* (β-casein) and *Wap* transcripts in total mammary gland mRNA samples from *Blimp1* mutants. The values were normalized to *Krt8* expression. Data are presented as mean values from *n*=2 mice of each genotype. (D) Quantification of pStat5^+^ cells from *Blimp1* mutants and littermate controls. Data are presented as mean±s.e.m. values from *n*=3 mice of each genotype. ***P<0.001. (E) Pups nursed by lactating WAP:Blimp1^cKO^ mutant females display growth retardation. Pups were weighed daily for 10 days. Body weights of pups are shown as mean±s.e.m. per litter per day, *n*=2 lactating females nursing identical litter sizes (*P*=0.0002). A total of 15 pups for each genotype were analysed. (F) Representative photographs of day 5 pups nursed by control and WAP:Blimp1^cKO^ females. Scale bars: 50 μm.

Consistent with these defects, immunostaining experiments together with qRT-PCR analysis revealed that expression of the milk components Wap and β-casein was significantly reduced (Fig. 8A-C). Stat5 phosphorylation in response to prolactin signalling promotes alveolar cell differentiation and induction of the milk protein gene signature (Liu et al., 1996). Nuclear phospho-Stat5 (pStat5) bound directly to milk gene promoters activates their expression (Jahchan et al., 2012). We found that Blimp1-deficient mammary glands display significantly reduced numbers of pStat5^+^ cells. However, Elf5 expression remains unaffected (Fig. 8A,B,D). Moreover, neonatal pups nursed by WAP:Blimp1^cKO^ females have less milk in their stomachs, and from birth onwards were growth retarded (Fig. 8E,F). Thus, Blimp1 is required for alveolar secretory function during pregnancy and lactation.

## DISCUSSION

Mammary gland development and tissue homeostasis depends on the combined activities of several distinct stem cell and progenitor populations (Visvader and Stingl, 2014). Here, we identify the zinc finger transcriptional repressor Blimp1 as a marker of a rare subset of luminal progenitors, initially present during puberty, that dramatically expand in response to pregnancy hormones giving rise to alveolar cells. Additionally, conditional inactivation experiments demonstrate that Blimp1 functional activity is required to promote ductal morphogenesis during puberty, and drive terminal differentiation of the milk-secreting alveoli during pregnancy.

It is well known that the luminal compartment comprises at least two distinct cell subsets. The mature ERα^+^ cell population seems to be mostly quiescent, with only rare proliferating cells detectable in the adult mammary gland (Russo et al., 1999; Shehata et al., 2012). Nonetheless, this population is essential for ductal morphogenesis (Bocchinfuso et al., 2000; Feng et al., 2007; Mallepell et al., 2006; Bernardo et al., 2010). Elf5 is widely viewed as an alveolar lineage-specific master regulator because Elf5 mutant mammary glands completely fail to initiate alveologenesis (Choi et al., 2009). The ERα^−^ luminal subpopulation, characterized by robust expression of the Ets transcription factor Elf5 (Oakes et al., 2008; Rios et al., 2014; Rodilla et al., 2015), is highly responsive to progesterone, which acts indirectly via RANKL signalling to upregulate Elf5 expression and promote alveolar cell expansion and functional maturation during pregnancy (Lee et al., 2013).

Here, we identify a novel subset of Blimp1^+^ERα^−^Elf5^+^ luminal progenitors. This rare but highly clonogenic subpopulation initially appears within the expanding ductal tree during puberty. However, at the onset of pregnancy this population expands dramatically. As for Elf5^+^ progenitors, Blimp1^+^ cells, representing <0.5% of the luminal population in virgin or pregnant females (Rios et al., 2014), possess a markedly elevated clonogenic capacity. Strikingly, in virgins 40% of isolated Blimp1^+^ luminal cells possess high self-renewal capacity and give rise to discrete colonies. At P18.5, >80% have clonogenic potential, compared with <10% for the Blimp1^−^ luminal cells. Blimp1 expression marks a unique population of progenitors that can contribute to both ductal and alveolar structures. The number of Blimp1^+^ progenitors within the epithelium dramatically increases in response to progesterone and oestrogen hormone treatment. Similarly, MEC cultures upregulate Blimp1 expression in response to RANKL stimulation. Thus, as for Elf5, Blimp1 is probably activated downstream of paracrine RANKL signalling from the adjacent ERα^+^PR^+^ luminal subpopulation. Interestingly, this RANKL-Nfatc1 signalling axis similarly induces Blimp1 expression during osteoclast differentiation (Nishikawa et al., 2010). Future work exploiting inducible labelling strategies will be necessary to define further the dynamic functional contribution made by this Blimp1^+^ cell lineage during successive rounds of pregnancy and lactation.

The present conditional inactivation experiments demonstrate Blimp1 functions at early stages during puberty to promote TEB remodelling (Mailleux et al., 2007). *Blimp1* mutant epithelium displays pronounced defects in lumen formation and ductal morphogenesis, associated with decreased proliferation and apoptosis. However, expansion of the ductal tree and induction of the alveolar buds during pregnancy proceeds normally. Rather, Blimp1 loss disrupts clearing of intraluminal and terminal differentiation of the alveoli responsible for milk production (Jahchan et al., 2012).

The establishment of epithelial polarity is required for functional maturation of alveolar cells (Choi et al., 2009; Elias et al., 2015; Jahchan et al., 2012). The present experiments uncover a requirement for Blimp1 in establishment of apical polarity. The Golgi apparatus is highly disorganized in mutant mammary gland tissue in both *in vivo* and *in vitro* Blimp1-depleted acini. Blimp1 is required to establish initial polarity as well as subsequent segregation of apical and basolateral compartments. Elf5 regulates expression of the tight junction and cell-cell adhesion molecules necessary during alveolar maturation (Choi et al., 2009).

Induction of milk synthesis by the alveolar cells occurs during pregnancy in response to prolactin-mediated activation of Stat5 (Miyoshi et al., 2001). pStat5 activation downstream of the prolactin receptor regulates expression of key milk protein components, including β-casein and Wap (Liu et al., 1995; Schmitt-Ney et al., 1991). *Stat5* mutants display defective lobuloalveolar development and lactation failure (Cui et al., 2004). Blimp1 conditional inactivation results in dramatically reduced Stat5 activity and impaired milk production. By contrast, loss of Blimp1 has no noticeable effect on Elf5 expression, also upstream of Stat5 activation (Choi et al., 2009). Rather, in the absence of Blimp1 here we observe defective luminal proliferation and polarization defects, suggesting that reduced Stat5 activation in *Blimp1* mutant cells reflects diminished receptor availability at the cell surface.

The present study identifies Blimp1 as a novel regulator of alveologenesis acting upstream to orchestrate secretory activation. In the B-cell lineage, Blimp1 functions as a master regulator governing terminal differentiation, and Blimp1 is both necessary and sufficient for maturation into immunoglobulin-secreting plasma cells (Martins and Calame, 2008). Thus, enforced Blimp1 overexpression drives mature B cells to become professional secretory cells. Similarly, here we have uncovered a key requirement for Blimp1 for maturation of the highly specialized milk-secreting cells of the mammary gland. It will be interesting to learn more about potentially common regulatory mechanisms governing Blimp1 functional roles in these professional secretory cell lineages.

Intriguingly, Blimp1 loss of function is accompanied by ectopic ERα expression in both the ductal and alveolar epithelium. Recent studies of human breast cancer cell lines suggest that Blimp1 directly represses ERα mRNA expression (Wang et al., 2009). However, this site was not identified in recent genome-wide Blimp1 chromatin immunoprecipitation analysis (Mould et al., 2015). Thus, the extent to which Blimp1 directly targets ERα expression *in vivo*, and possibly contributes to governing the balance of ERα^+^ versus ERα^−^ cell populations during mammary gland homeostasis and remodelling, remains unknown. Poorly differentiated breast cancers are thought to arise from ERα^−^ luminal progenitors (Molyneux et al., 2010; Pratt et al., 2009). Here, we identified Blimp1 as a novel marker for luminal ERα^−^ progenitors. Given the established link between Blimp1 and EMT in breast cancer, unravelling the mechanisms that underlie Blimp1 functions in mammary gland development should not only yield important insights into stem cell/progenitor cell populations in the mammary epithelium but also contribute to a better understanding of breast cancer biology.

## MATERIALS AND METHODS

### Animals

C57BL/6 mice were used as the wild-type strain. The BAC transgenic reporter strain expressing membrane-targeted Venus under the control of the Blimp1 regulatory elements (Prdm1-mVenus; BV) has been described (Ohinata et al., 2008). For tissue-specific deletion experiments, *K14-Cre* (Vasioukhin et al., 1999) or *WAPi-Cre* (Wintermantel et al., 2002) animals were intercrossed with *Prdm1^BEH/+^* (Vincent et al., 2005) animals to generate heterozygous *K14-Cre*,*Prdm1^BEH/+^* or *WAPi-Cre*,*Prdm1^BEH/+^* males that were then mated with homozygous *Prdm1^CA/CA^* females (Shapiro-Shelef et al., 2003) to generate control (*Prdm1^CA/+^*, *WAPi-Cre* or *K14-Cre*), heterozygous (*K14-Cre*,*Prdm1^CA/+^*; *WAPi-Cre*,*Prdm1^CA/+^*, or *Prdm1^BEH/+^*) or null (*WAPi-Cre;Prdm1^CA/BEH^* or *K14-Cre;Prdm1^CA/BEH^*) females (referred to as K14:Blimp1^cKO^ and WAP:Blimp1^cKO^, respectively). For *Blimp1* gene deletion in mammary epithelial culture, *Prdm1^BEH/+^* and *Prdm1^CA/CA^* mice were crossed with the *ROSA26:Cre^ERT2^* line (Vooijs et al., 2001) to generate *Prdm1^BEH/CA^;ROSA26:Cre^ERT2^* females (referred to as Blimp1^cKO^). Mice were genotyped by PCR as described in the original reports. All animal experiments were performed in accordance with Home Office (UK) regulations and approved by the University of Oxford Local Ethical Committee.

### Ovariectomy and hormone treatment

Wild-type and *Prdm1^Cre.IRES.LacZ^* 8- to 10-week-old female mice were bilaterally ovariectomized and allowed to recover for 10 days. Mice were divided into two experimental groups (control and hormone treated), and injected subcutaneously with 100 µl mineral oil alone or containing 10 µg 17β-oestradiol (E2) (Sigma) plus 1 mg progesterone (Pg) (Sigma) (E2+Pg) as previously described (Joshi et al., 2010). Mammary tissues were stained for Carmine and X-gal as described previously (Elias et al., 2014).

### Whole mounts and quantification of ductal morphogenesis

For whole-mount Carmine staining, inguinal mammary glands were fixed overnight with Carnoy's solution (60% methanol, 30% chloroform and 10% glacial acetic acid), hydrated in an ethanol series, stained with Carmine Alum (1% Carmine, 2.5% aluminum; Sigma, UK), then dehydrated in ethanol and cleared with Histo-clear II (National Diagnostics) as described previously (Elias et al., 2014).

Mammary gland development was quantified as described elsewhere (Elias et al., 2015). In brief, the degree of ductal invasion was determined by dividing the duct length by the mammary gland length from the mid-point of the lymph node, and the numbers of total branches and TEBs were determined on whole-mount images using ImageJ (http://rsb.info.nih.gov/ij/; NIH, US).

### Histological analysis and immunostaining

Mammary glands were dissected, fixed overnight in 4% paraformaldehyde (PFA), embedded in paraffin, sectioned (6 μm) and stained with Haematoxylin and Eosin (H&E) for histological analysis. For immunostaining, paraffin sections (6 μm) were deparaffinized, rehydrated and subjected to antigen retrieval by boiling for 1 h in either sodium citrate, pH 6.0 (Wap, β-casein, ErbB2) or Tris-EDTA pH 9.0 [Krt8, Ki67, p63 (also known as Trp63), cleaved caspase-3, oestrogen receptor α (ERα), Blimp1, Par3, podocalyxin and GM130] (Table S1). Sections were incubated overnight at 4°C in primary antibodies, washed in PBS, developed using the appropriate Envision peroxidase-labelled polymer kit (Dako) and counterstained with Haematoxylin. For double immunofluorescence, paraffin sections were antigen retrieved as described above followed by application of primary antibodies (Table S1). Alternatively, PFA-fixed frozen sections (30-50 µm) were cut, air-dried for 30 min, permeabilized for 45 min in PBS/0.2% Triton X-100, and incubated for 2 h in BSA/FBS block [2% bovine serum albumin (BSA), 5% fetal bovine serum (FBS), 0.2% Triton X-100 in PBS]. Sections were incubated overnight at 4°C with primary antibodies, followed by incubation for 1 h at room temperature with secondary antibodies. Secondary antibodies were conjugated to Alexa Fluor 488 or Alexa Fluor 594 (Life Technologies). Sections were counterstained with DAPI-containing Fluoroshield (Sigma). Immunofluorescence images were captured with an inverted Olympus FV1000 laser scanning confocal microscope equipped with ×40 and ×60 oil-immersion objectives. *z*-stacks were collected at 1-μm intervals. Images were analysed with ImageJ. A full description of antibodies is provided in the Table S1.

### BrdU pulse-chase experiments and apoptotic cell analysis in TEBs

To assess cell proliferation, 0.25 mg 5-bromo-2-deoxyuridine (BrdU, Sigma) per gram of body weight was injected intraperitoneally 2 h prior to sacrifice. Mammary glands fixed in 4% PFA were paraffin embedded, sectioned (6 μm) and stained using anti-Krt8 and BrdU antibodies. The numbers of BrdU^+^ cells were counted as a percentage of total number of cells within each TEB of 5- and 6-week-old virgin mice. A minimum of ten TEBs from three separate mice (∼2-3×10^3^ cells) were counted per genotype. To assess cell death, paraffin sections were assessed for CC-3. The percentage of immunoreactive cells within TEBs was determined from a minimum of ten TEBs, from three separate mice (∼10^4^ cells) per genotype, as described previously (Wiseman et al., 2003).

### X-gal staining

Mammary glands were fixed in 1% PFA overnight at 4°C, washed in PBS and stained overnight at 37°C in X-gal staining solution [0.5 mg/ml X-gal, 5 mM K_3_Fe(CN)_6_, 5 mM K_4_Fe(CN)_6_, 5 mM ethylene glycol tetra-acetic acid (EGTA), 2 mM MgCl_2_, 10% NP-40, 0.01% deoxycholic acid]. Tissue was then dehydrated, embedded in paraffin, sectioned (6 μm) and counterstained with Nuclear Fast Red.

### Isolation of mammary epithelial cells and flow cytometry

Mammary epithelial cells (MECs) and the separation of basal and luminal cells were performed as described elsewhere (Stingl et al., 2006; Taddei et al., 2008). Once mechanically dissociated, mammary fat pads were digested (90 min, 37°C) in CO_2_-independent medium (Invitrogen) containing 5% FBS, 3 mg/ml collagenase A (Roche Diagnostics) and 100 U/ml hyaluronidase (Sigma). Cells were resuspended in 0.25% trypsin-EDTA (1 min), and then in 5 mg/ml dispase (Roche Diagnostics) with 0.1 mg/ml DNase I (Sigma) (5 min). Red blood cells were lysed with 0.17 mM NH_4_Cl. Basal and luminal cells were isolated from mammary epithelial cells obtained from the inguinal glands. Cells were stained with the following antibodies: CD24-PerCP-Cy5.5 (clone M1/69; BD Pharmingen), CD49f-PE (clone GoH3; BD Pharmingen), CD45-APC (clone 30-F11; Biolegend) and CD31-APC (clone MEC13.3; Biolegend). Basal (CD24-low/CD49f-high) and luminal (CD24-high/CD49f-low) cells were purified using MoFlo XDP Flow Cytometer (Beckman Coulter).

### Mammary colony-forming assays

*In vitro* Matrigel colony-forming assays were performed as described (Stingl et al., 2006). Single mammary cell suspensions were prepared from the inguinal glands and 3000 unsorted cells or sorted luminal cells were seeded in 50 µl Matrigel and cultured in DMEM/F12 medium containing 1% FBS (HyClone Laboratories) and B27 (Life Technologies). Colonies were scored after 6 days.

### Primary cell 3D culture, gene deletion, immunofluorescence and transmission electron microscopy (TEM)

Primary MECs were collected from 15.5- and 16.5-day pregnant mice and cultured as described (Akhtar and Streuli, 2013). Cells were seeded onto Matrigel (BD Biosciences) to form acini and cultured in growth media [DMEM/F12 medium (Life Technologies) containing 5 µg/ml insulin, 1 µg/ml hydrocortisone (Sigma), 3 ng/ml epidermal growth factor (EGF), 10% FBS, 50 U/ml penicillin/streptomycin, 0.25 µg/ml fungizone and 50 µg/ml gentamycin]. Cells (2×10^4^) were plated in each well of 8-well LabTek II chamber slides (Thermo Fisher Scientific) pre-coated with Matrigel (25 μl per well). Cells were fed every 48 h and grown for 3-10 days. Cre-mediated deletion of Blimp1 in primary MEC cultures was achieved by collecting MECs from wild-type or *Prdm1^BEH/CA^;ROSA26:Cre^ERT2^* mice and treating with 100 nM 4OHT (Sigma) dissolved in ethanol for 24 h.

MEC cultures derived from *Prdm1^Cre.IRES.LacZ^* mice were incubated with or without 200 ng/ml murine RANKL (PeproTech) for 24 or 48 h, followed by X-gal staining as described above.

Acini were fixed with 4% PFA and permeabilized with 0.5% Triton X-100 in PBS. Fixed cells were blocked with 10% normal goat serum/2% BSA in PBS for 2 h, and then incubated with Blimp1, F-actin, cleaved caspase-3, Par3, GM130 and Ki67 antibodies (Table S1) overnight at 4°C. Cells were stained with mouse and rabbit Alexa Fluor 488- or Alexa Fluor 555-conjugated antibodies. Acini with F-actin staining at the apical surface of cells surrounding a single lumen were identified as acini with normal lumens. For all immunostaining, slides were mounted in DAPI-containing Fluoroshield. The pictures were captured with an inverted Olympus FV1000 laser scanning confocal microscope equipped with ×40 and ×60 oil-immersion objectives. *z*-stack steps were of 1 μm. Images were analysed with ImageJ.

For TEM analysis, day 6 acini were first fixed with 4% PFA plus 2.5% glutaraldehyde in 0.1 M PIPES buffer (pH 7.2; Sigma) for 2 h at room temperature, and then overnight at 4°C. Samples were washed with 0.1 M PIPES buffer and incubated in 1% osmium tetroxide (OsO_4_; TAAB Laboratories) in 0.1 M PIPES buffer at 4°C for 2 h with gentle rotation. The samples were washed with water, then *en bloc* stained with 0.5% aqueous uranyl acetate (TAAB Laboratories) overnight at 4°C in the dark. The samples were gradually dehydrated in ethanol at 4°C (15 min each with 30%, 70% 50%, 80%, 90%, 95%, 100% ethanol), infiltrated with TAAB TLV resin over 3 days, then polymerized overnight at 60°C. Ultrathin (90 nm) sections were taken using a Diatome diamond knife on a Leica Ultracut7 ultramicrotome. Sections were post-stained with lead citrate for 5 min, then examined on a Tecnai 12 transmission electron microscope (FEI) equipped with a Gatan OneView CMOS camera.

### Quantification and image analyses

To measure relative fluorescence intensity at the apical surface, a 30-pixel line was drawn across the apical surface and the cytoplasm using ImageJ software. The line scan function of ImageJ was used to reveal the relative fluorescence intensity across the line. To measure Golgi orientation in 3D culture, we used a macro developed for ImageJ as described previously (Elias et al., 2015).

### RNA extraction and qRT-PCR

Total RNA was extracted from MECs obtained by FACS sorting, 3D MEC cultures or from digested whole-mount mammary glands using the RNeasy Mini Kit (Qiagen). RNA (1 μg) was used to synthesize first-strand cDNA (Invitrogen) followed by real-time PCR using QuantiTech SYBR Green master mix (Q2040143; Qiagen) on a Rotor-Gene Q (Qiagen). Primer sequences are provided in Table S2.

### Statistical analyses

GraphPad Prism 6.0 software was used for statistical analysis. Data are shown as mean±standard error of the mean (s.e.m.). Student's *t*-test was used where applicable, with *P*<0.05 considered significant. Comparison of body weights of pups suckled by *Blimp1* mutant or wild-type females was analysed by two-way ANOVA with repeated measures.
